# Waist Circumference Independently Associates with the Risk of Insulin Resistance and Type 2 Diabetes in Mexican American Families

**DOI:** 10.1371/journal.pone.0059153

**Published:** 2013-03-11

**Authors:** Manju Mamtani, Hemant Kulkarni, Thomas D. Dyer, Laura Almasy, Michael C. Mahaney, Ravindranath Duggirala, Anthony G. Comuzzie, John Blangero, Joanne E. Curran

**Affiliations:** Department of Genetics, Texas Biomedical Research Institute, San Antonio, Texas, United States of America; Sapienza, University, Italy

## Abstract

**Objective:**

In spite of the growing recognition of the specific association of waist circumference (WC) with type 2 diabetes (T2D) and insulin resistance (IR), current guidelines still use body mass index (BMI) as a tool of choice. Our objective was to determine whether WC is a better T2D predictor than BMI in family-based settings.

**Research Design and Methods:**

Using prospectively collected data on 808 individuals from 42 extended Mexican American families representing 7617.92 person-years follow-up, we examined the performance of WC and BMI as predictors of cumulative and incident risk of T2D. We used robust statistical methods that accounted for the kinships and included polygenic models, discrete trait modeling, Akaike information criterion, odds ratio (OR), relative risk (RR) and Kullback-Leibler R^2^. SOLAR software was used to conduct all the data analyses.

**Results:**

We found that in multivariate polygenic models, WC was an independent predictor of cumulative (OR = 2.76, p = 0.0002) and future risk of T2D (RR = 2.15, p = 3.56×10^−9^) and outperformed BMI when compared in a head-to-head fashion. High WC (≥94.65 cm after adjusting for age and sex) was also associated with high fasting glucose, insulin and triglyceride levels and low high-density lipoprotein levels indicating a potential association with IR. Moreover, WC was specifically and significantly associated with insulin resistant T2D (OR = 4.83, p = 1.01×10^−13^).

**Conclusions:**

Our results demonstrate the value of using WC as a screening tool of choice for future risk of T2D in Mexican American families. Also, WC is specifically associated with insulin resistant T2D.

## Introduction

The prevalence of Type 2 diabetes (T2D) is rapidly increasing worldwide.1–3] This upsurge is concomitant with the sudden global increase in the prevalence of obesity, an established risk factor in the pathogenesis of T2D.4–7] This concomitance indicates that the operationally easy-to-measure and accurate anthropometric indexes that characterize obesity may also closely associate with the risk of T2D and may therefore be used for the screening of T2D. Such a public health intervention can be expected to augment the programmatic yield of T2D detection strategies and provide more opportunities for effective prevention and control of T2D. Indeed, current guidelines by various agencies like the WHO, ADA and German Diabetes Society (Deutsche Diabetes Gesellschaft, DDG) recommend body mass index (BMI) as the primary screening anthropometric index for T2D.8]

There is now a growing recognition that central rather than general obesity is more contributory to and therefore better correlates with the risk of T2D.9–13] Interestingly, BMI is an indicator of generalized obesity while waist circumference (WC) shows an excellent correlation with central obesity.13,14] Thus, WC should be theoretically more useful than BMI to predict the risk of T2D. In this context, we 15] and others have demonstrated the superiority of WC over BMI for screening of T2D in epidemiological settings.16–19] Such a paradigmatic shift from the use of BMI to WC for screening of T2D entails that the screening efficacy of WC should also be demonstrated in other settings such as family studies. This is important since WC has been shown to be a highly heritable trait 20–22]. In this regard, Gao et al. 23] have recently shown that using WC as a monitoring tool for T2D may be beneficial in family settings. Additional studies are required that robustly demonstrate the screening performance of WC in family studies across the world. Further, the relative importance of BMI and WC in screening for T2D risk in the families is unknown.

We conducted this study with the following research questions: 1) Is WC associated with an increased risk of current or future T2D and its related traits in pedigreed Mexican American individuals; and 2) Is WC better than BMI for predicting T2D risk? To answer these two questions, we used the rich resource of Mexican American subjects enrolled in the San Antonio Family Heart Study (SAFHS) and examined the absolute and relative performance of WC for the prediction of T2D in family settings.

## Materials and Methods

### Ethics Statement

Informed written consent was obtained from all participants before collection of samples. The Institutional Review Board of the University of Texas Health Sciences Center at San Antonio approved the study.

### Study subjects

The SAFHS is an ongoing endeavor that focuses on 1,431 members of 42 large and extended Mexican American families in San Antonio. Details of this study have been described elsewhere.24,25] Briefly, this collaborative research effort involving the Texas Biomedical Research Institute and the University of Texas Health Science Center at San Antonio began in 1991 and currently includes data on ∼2000 individuals. The SAFHS aims to quantify the relative contributions of genetic and environmental factors to the risk of developing cardiovascular diseases and metabolic syndrome. Extensive phenotypic assessment for a number of traits related to metabolic syndrome has been performed in these individuals. As a part of this study, the participants were also followed up prospectively. Currently data for the baseline and two follow-up visits is available. We used this prospectively collected data for the longitudinal component of this study.

### Outcomes and predictors

We studied three primary outcomes: cumulative risk of T2D, risk of incident T2D and risk of future insulin resistance (IR). Cumulative risk of T2D was defined as concurrent existence or future development of T2D. T2D was diagnosed according to American Diabetes Association criteria.26] Participants who reported to be under treatment with either oral anti-diabetic agents or insulin, or who gave a history of diabetes were also considered to have T2D. Incident T2D was defined as detection of new cases of T2D during follow-up. IR was measured by the Homeostasis Model of Assessment–Insulin Resistance (HOMA-IR). The HOMA-IR was estimated as follows – fasting glucose (mmol/L) x fasting insulin (µU/ml)/22.5.27] For defining IR we used HOMA-IR cut-points of 2.6 (the commonly used clinical cut-point for IR) and 3.8 (as specifically recommended for Mexican-American populations).28] We also examined associations with several T2D-related traits as secondary outcomes. These included fasting glucose, fasting glucose adjusted for anti-diabetic drug use, serum insulin, triglycerides, total serum cholesterol, high density lipoprotein cholesterol (HDL-C), directly measured low density lipoprotein cholesterol (LDL-C), LDL-C fraction 1 and LDL-C fraction 2. Blood samples were obtained after a 12-hour fast for measurement of various phenotypes including glucose, total cholesterol, triglycerides, LDL and HDL cholesterol, and they were collected again 2 h after a standardized oral glucose load to measure plasma glucose. All the secondary outcomes and insulin resistance were assessed prospectively at the second follow-up visit.

We examined the association of the following 15 anthropometric indexes with one or more of the aforementioned outcomes. The anthropometric indexes included skin-fold thicknesses (biceps, triceps, forearm, subscapular, abdominal, suprailiac, medial calf and lateral calf), waist and hip circumferences, weight, height and three composite indexes: BMI, waist/hip ratio (WHR) and subscapular/triceps ratio (STR). Methods for measurements of these indexes have been described previously.22,25]

### Statistical analysis

We used univariate and multivariate polygenic models to study the association of various anthropometric indexes with cumulative risk of T2D. All the polygenic models used in this study were of the form:
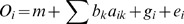



where, O is the outcome of interest; m is the trait mean; a is the covariate vector of dimension k with b as the corresponding regression coefficients; g is the polygenic effect and e is the residual error for an individual indexed by i. In all of these models we included age, age^2^, age*sex, age^2^*sex and sex as covariates. For univariate analyses the polygenic models included the abovementioned covariates and each anthropometric index separately. Model fits were compared using log-likelihoods (for all outcomes) and the Kullback-Leibler R^2^ (K-L R^2^, for dichotomous outcomes only). For multivariate analyses, all the anthropometric indexes were simultaneously included in a single model along with the abovementioned covariates. However, since WHR is highly correlated with WC by definition, we could not use a single multivariate model including all the composite indexes (BMI, WHR and STR) as covariates. Instead, we ran univariate polygenic models for each of these indexes and then compared the model fits using K-L R^2^. For comparing regression coefficients of different indexes, we estimated the standardized regression coefficient for each index as the regression coefficient divided by its standard error.

Statistical significance of the regression coefficient estimated from a polygenic model was determined by constraining the regression coefficient to zero, estimating the difference in log-likelihoods between the constrained and unconstrained models and applying a chi-square test. For dichotomous outcomes, the discrete trait modeling procedure was used. The odds ratio (OR) of a dichotomous outcome was determined as 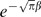 since the SOLAR software 29] returns a negative regression coefficient from a probit model for a positively associated covariate.

For ease of clinical usage, we estimated the optimal cut-point for WC as a predictor of the cumulative risk of T2D. We dichotomized WC by sliding cut-points over the observed range. At each cut-point we used age and sex adjusted polygenic models with the dichotomized WC as a covariate. From each model we determined the Akaike Information Criterion (AIC) [estimated as -2×Loglikelihood – 2*(number of fitted parameters)] and the OR. The optimal cut-point was estimated as that at which the AIC was minimum and OR was maximum. SOLAR software was employed in all the statistical analyses and statistical significance was assessed at a type I error rate of 0.05.

## Results

### Study subjects

We studied 808 participants from 42 extended Mexican American families on whom data for various metabolic, anthropometric and demographic variables was available. One hundred and seventy eight (22.02%) subjects developed T2D by visit 3 of whom 100 subjects were detected as new diabetes cases during follow-up. The total length of follow-up was 7617.92 person-years, translating to an incidence rate of 13.13 T2D cases/1000 population/year. The mean age of the study sample was 37.0 (SD = 14.39) years and there were 292 (36.1%) males. Prevalence of insulin resistance was 74.6% based on a HOMA-IR cut-off of 2.6 and 56.1% using a cut-off of 3.8.

### Anthropometric indexes and cumulative risk of T2D

We first studied the associations of various anthropometric indexes like skin-fold thicknesses (biceps, triceps, forearm, subscapular, abdominal, suprailiac, medial calf and lateral calf), waist and hip circumferences, and weight and height with the cumulative risk of T2D (Table 1). We used univariate and multivariate polygenic regression models adjusting for age, sex and their interaction to evaluate these associations. In univariate polygenic analyses, all the anthropometric indexes except lateral calf thickness and height were independently and significantly associated with the cumulative risk of T2D (Table 1). Interestingly, WC showed the strongest association with the cumulative risk of T2D (β = −0.4761, p = 4.30×10^−14^). In the multivariate polygenic model (i.e. including all the anthropometric indexes in the single model), we observed that only biceps skinfold thickness, lateral calf skinfold thickness and WC were significantly associated with the cumulative risk of T2D (Table 1). Notably, the strength of association of WC with cumulative risk of T2D increased in the multivariate context as compared to that in the univariate context (β = −0.5746 and −0.4761; OR = 2.76 and 2.32 for multivariate and univariate models, respectively).

**Table 1:** Univariate and multivariate association of anthropometric indexes with cumulative risk of T2D.

Anthropometric Index	Univariate Analysis			Multivariate Analysis		
	B	OR (95% CI)	P	β	OR (95% CI)	P
Skinfold Thickness						
Biceps	−0.3795	1.96 (1.52–2.52)	5.83×10^−9^	−0.3842	1.97 (1.19–3.28)	0.0083
Forearm	−0.1795	1.37 (1.10–1.71)	0.0041	0.1711	0.74 (0.48–1.13)	0.1588
Triceps	−0.2345	1.51 (1.18–1.94)	0.0007	0.0999	0.84 (0.53–1.33)	0.4469
Subscapular	−0.3420	1.83 (1.47–2.29)	2.75×10^−8^	−0.0925	1.18 (0.79–1.76)	0.4210
Abdominal	−0.3072	1.72 (1.36–2.17)	6.79×10^−7^	0.1790	0.73 (0.47–1.14)	0.1560
Suprailiac	−0.3949	2.01 (1.59–2.54)	4.38×10^−10^	−0.1341	1.27 (0.76–2.12)	0.3549
Medail Calf	−0.1672	1.34 (1.08–1.67)	0.0073	−0.0456	1.08 (0.69–1.69)	0.7196
Lateral Calf	0.0117	0.98 (0.94–1.02)	0.851	0.3676	0.52 (0.35–0.77)	0.0008
Circumferences						
Waist	−0.4761	2.32 (1.84–2.93)	4.30×10^−14^	−0.5746	2.76 (1.59–4.81)	0.0002
Hip	−0.3410	1.83 (1.46–2.30)	2.89×10^−8^	0.2734	0.62 (0.33–1.16)	0.1232
Others						
Weight	−0.3828	1.97 (1.58–2.46)	4.52×10^−10^	−0.2439	1.54 (0.78–3.06)	0.2144
Height	0.0208	0.96 (0.72–1.29)	0.8061	0.1595	0.75 (0.51–1.11)	0.1501

β, regression coefficient; OR, odds ratio; CI, confidence interval; p, significance value

### WC as a predictor of future T2D

As WC was strongly associated with the cumulative risk of T2D, we next assessed whether WC can also predict the future risk of T2D. For this we studied the association of WC with the incident cases of T2D (new cases during follow-up) using the polygenic model. Indeed, WC was also significantly associated with an increased risk of incident T2D [β = −0.43, RR (95% CI) = 2.15 (1.64–2.82) p = 3.56×10^−9^]. Thus WC was not only associated with the cumulative risk of T2D but also predicted the future risk of T2D.

### Comparison of WC with composite anthropometric indexes

Subsequently, we compared the performance of WC with other composite anthropometric indexes like BMI, WHR and STR for predicting the cumulative risk of T2D using univariate polygenic models. We observed that the K-L R^2^ values for WC, BMI, WHR and STR were 0.20, 0.18, 0.19 and 0.13, respectively. The standardized regression coefficients (p) for these indexes were −7.1034 (4.20×10^−14^), −6.3093 (2.44×10^−11^), −6.5005 (9.17×10^−13^) and −2.2081 (0.0456), respectively. Importantly, WC was strongly correlated with the cumulative T2D risk. However, the heritability of WC was marginally lower than that of BMI (0.54 versus 0.56).

To address our second research question, we further compared the associative performance of WC and BMI in a head-to-head fashion. In a multivariate polygenic model we included both WC and BMI as correlates of cumulative T2D risk. To ensure that these analyses were not influenced by potential collinearity between WC and BMI, we first estimated the total phenotypic correlation between these two traits. For this reason, we used bivariate trait analyses in which the kinship structure, age, sex and their interactions were accounted for. We found that 23.29% of the model variance was unique and not accounted for by the phenotypic correlation between WC and BMI, thus mitigating the possible influence of collinearity on our results. We estimated from the polygenic model that WC (standardized β = −3.67, p = 0.0002) was a more powerful predictor of cumulative T2D risk than BMI (standardized β = −0.09, p = 0.9269). Moreover, when we compared the performance of WC and BMI for predicting the future risk of T2D in a multivariate polygenic model, we observed that WC [standardized β = −7.29, RR (95% CI) = 2.12 (1.65–2.71) p = 0.0066] was a better and stronger predictor of incident T2D than BMI [standardized β = −0.41, RR (95% CI) = 1.05 (0.84–1.32) p = 0.8480].

### Determination of the optimal cut-point for WC

**Figure 1:**
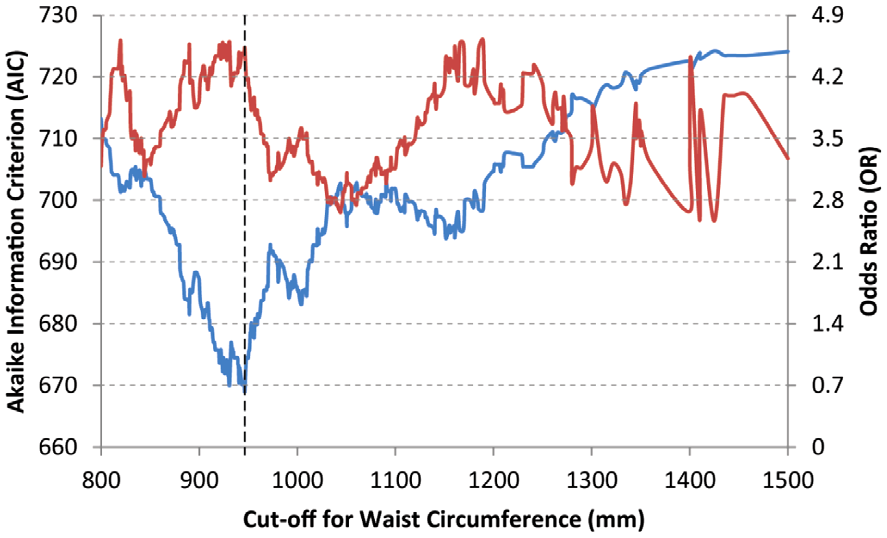
Determination of the optimal cut-point for waist circumference as a predictor of cumulative T2D risk. Figure shows Akaike information criterion (left y-axis) and odds ratio (right y-axis) for a cut-point of waist circumference indicated on the x-axis. Dashed vertical line indicates the optimal cut-point.

We aimed to find an age- and sex-adjusted cut-point that can simply yet informatively dichotomize WC as a correlate of the cumulative T2D risk. The best cut-point (as indicated by the minimum AIC of 669.05) in this population was 94.65 cm (Figure 1). At this cut-point the OR (95% CI) for cumulative T2D risk was 4.53 (2.98–6.87). Interestingly, another peak in OR was observed at a WC cut-point of 118.5 cm. While this peak could be construed as representing a cut-point for males, we found that the AIC at this cut-off was quite high (698.55) as compared to that for the gender-nonspecific cut-point of 94.65 cm.

Arguably, use of a gender-agnostic cut-point may lose diagnostic information as compared to the strategy of using a gender-specific cut-point. To directly contrast these two strategies, we compared the predictive performance of the gender-agnostic cut-point with that of the recommended gender-specific WC cut-points for the US population (≥102cm for males and ≥88 cm for females).30] We found that the strategy of using a single cut-point demonstrated predictive performance comparable to the strategy of using the gender-specific cut-points (AIC of 669.05 versus 658.25, that is an information loss of 1.6% due to gender-agnostic cut-point). Moreover, the OR for T2D risk associated with the single cut-point strategy (4.53) was better than that associated with the strategy of gender-specific cut-points (3.98).

### Association of dichotomized WC with T2D-related traits

**Figure 2:**
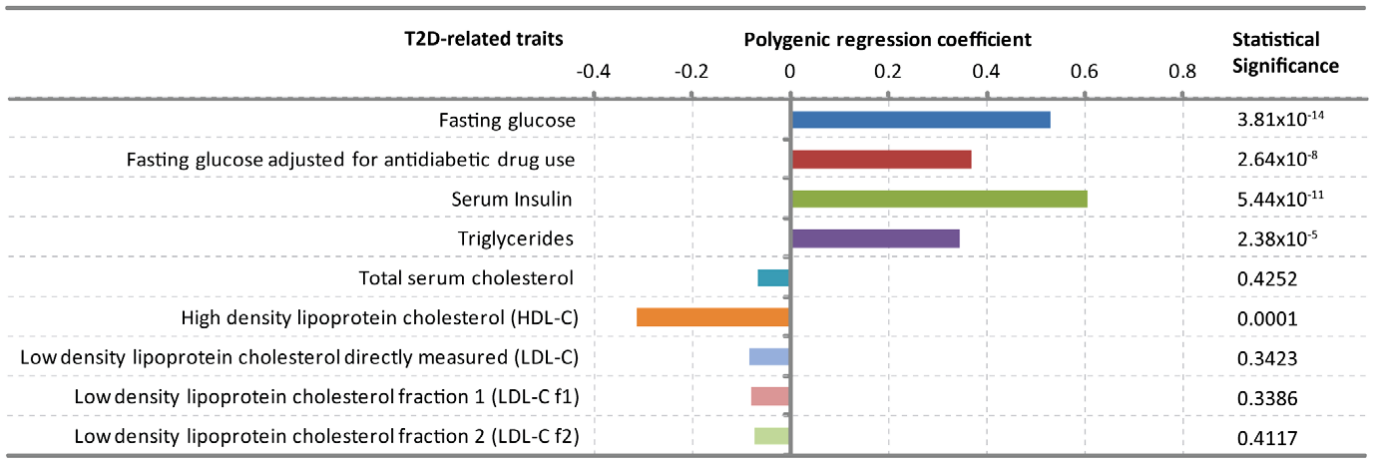
Association of dichotomized WC with T2D-related traits. The bars represent regression coefficients estimated using polygenic regression models.

We observed that dichotomized WC was significantly associated with high fasting glucose (β = 0.5251, p = 3.81×10^−14^), fasting glucose adjusted for anti-diabetic drug use (β = 0.3659, p = 2.64×10^−8^), high serum insulin (β = 0.6025, p = 5.44×10^−11^), high triglycerides (β = 0.3422, p = 2.38×10^−5^) and low HDL-C (β = −0.3142, p = 0.0001) (Figure 2). Only 1.8% of study subjects were receiving lipid-lowering drugs, and adjustment for the use of these drugs did not alter the results significantly (Table S1).

### Association of dichotomized WC with IR and insulin resistant T2D

**Figure 3:**
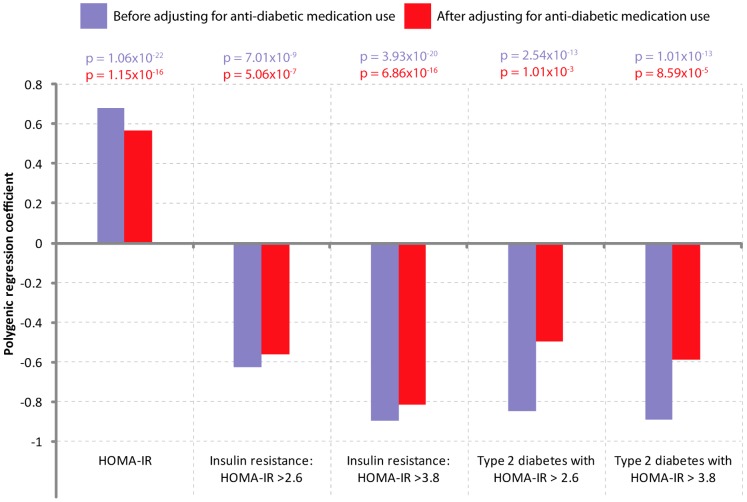
Association of dichotomized WC with IR and insulin resistant T2D. The bars represent regression coefficients estimated using polygenic regression models. Results are shown before (purple bars) and after (red bars) adjusting for the use of antidiabetic medication which includes the oral antidiabetic drugs as well as insulin. Statistical significance of a regression coefficient is shown in color-coded fashion at the top of the graph.

The abovementioned associations are interesting because high fasting glucose, high serum insulin, high triglycerides and low HDL-C are all indicators of insulin resistance.31,32] Therefore, we next assessed whether dichotomized WC is associated with IR in general and insulin resistant T2D (defined as presence of IR as well as T2D) in particular. We observed that dichotomized WC was highly predictive of both IR and insulin resistant T2D (Figure 3). Interestingly, dichotomized WC strongly predicted T2D with HOMA-IR >3.8 [β = −0.8902, OR (95% CI) = 4.83 (3.12–7.49), p = 1.01×10^−13^, Figure3].

The definition of HOMA-IR used in our study did not consider the concurrent use of anti-diabetic agents. To safeguard against the potential confounding due to this, we repeated the abovementioned analyses by adjusting for the use of anti-diabetic drugs. Our results still concurred with earlier interpretations (compare the purple and red bars in Figure 3).

## Discussion

Our results clearly demonstrate that WC is the strongest anthropometric index that associates with insulin resistance and T2D in Mexican American families whether examined longitudinally or cumulatively. Irrespective of age and gender, WC exceeding 94.65 cm was most informative with regard to a cumulative risk of T2D. Of note, in univariate or multivariate contexts, WC was more strongly related to cumulative or incident risk of T2D as compared to BMI. The implications of our results need to be considered in the light of three important aspects of research related to metabolic syndrome and T2D.

First, there is an ongoing debate on the use of WC or BMI in screening programs for early detection of T2D.8,33] Various clinical guidelines primarily favor the use of BMI in screening programs, while a recent meta-analysis 33] indicates that BMI and WC can be used interchangeably since they have similar predicting abilities for future risk of T2D. On the other hand, there is now a growing recognition that WC may be more suited than BMI as a predictor of T2D risk.34] Studies in various populations 15,18,35] have demonstrated the superiority of WC over BMI in this regard. Our findings support the view that WC should be used in screening programs instead of BMI because 1) WC is strongly associated with the risk of both prevalent and incident T2D; 2) WC is also an indicator of insulin resistance (irrespective of the presence of T2D) and insulin resistant T2D (i.e. insulin resistance with the presence of T2D); 3) In a single multivariate model WC outperformed BMI as a predictor of cumulative as well as incident risk of T2D; and 4) In spite of its high heritability WC still independently predicted the risk of T2D in pedigreed Mexican American families. Moreover, WC is as simple, convenient, inexpensive and easy to use in clinical practice as BMI and it can be easily monitored by patients themselves. For these reasons we believe that reevaluation of existing guidelines for screening of T2D is needed.

Second, the role of WC for prediction of T2D risk in families has been understudied. To our knowledge, a prospective evaluation of the importance of WC in diabetes pathogenesis in extended pedigrees has not been studied. Our results therefore proffer novel evidence in that regard. Since use of families as units can improve outcomes of diabetes screening programs,36] our findings point towards the possibility of further refining such strategies by inclusion of WC as the primary screen. WC is one of the requirements for the diagnosis of metabolic syndrome and the International Diabetes Federation (IDF) recommends that WC cut-points specific for different populations are needed.30] Our results demonstrate that an age-, sex-adjusted cut-point of 94.65 cm was highly informative in this ethnic population. While the generalizability of this cut-point remains limited, it is noteworthy that the optimal WC cut-point observed in this study for the prediction of T2D risk is practically close to the average of the recommended gender specific cut-points (102 cm for males and 88 cm for females) for the diagnosis of metabolic syndrome.30] This would therefore indicate that the recommended WC cut-point for the diagnosis of metabolic syndrome might also be useful for predicting the future risk of T2D. Further, our results suggest that use of a single population-specific WC cut-point may be at least as informative as gender-specific cut-points.

Third, we found that WC was specifically associated with future risk of insulin resistance as well as insulin resistant T2D. WC is associated with IR since it closely associates with visceral obesity, which is a critical determinant of IR. Indeed, WC in itself is considered to be a strong predictor of visceral fat.12] Mechanistically, increased secretion of free fatty acids and inflammatory cytokines combined with decreased secretion of adiponectin orchestrate in the multivariate culmination in visceral obesity and insulin resistance.37] At the level of the adipocyte, hyperinsulinaemia characteristic of IR activates 11-hydroxysteroid dehydrogenase in the omental adipose tissue and is followed by release of active cortisol. These changes induce a cushingoid fat distribution and increase in WC.38] Our observations afford a strong support to these biological underpinnings. Recent past has seen an accretion of epidemiological evidence that bolsters the associative/causal link between WC and IR. However, prospective family-based studies that show such a link have generally been lacking.

The clear strengths of our approach are a family-based prospective study design, an ethnically homogenous sample, a large sample size, extensive follow-up data and robust statistical methods. However, our study suffers from limitations inherent in any observational study of this type. First, the attrition rate in the present study was 33.1%, which is slightly higher than that seen in typical prospective studies. Since information on metabolic syndrome related traits was not available for the individuals who did not complete the follow up, it was not possible to predict the direction of effect of this potential attrition bias on the strength of tested associations. However, assuming that the attrition pattern was missing-at-random, we believe that the attrition would only under-power our interpretations and not bias them either-way. Second, due to the nature of the periodically scheduled visits, the exact time of event (i.e. the date of T2D occurrence) in the study participants is unknown. Instead, we used the cumulative risk of T2D as our primary outcome. This outcome variable captures the existing and prospective risk of T2D development. Lastly, our study sample represents a high risk population for metabolic syndrome and therefore these results cannot be directly applied to the general population.

Notwithstanding these limitations, our results provide compelling support to the burgeoning notion that WC is a simple and accurate predictor of ensuing and impending T2D especially if IR is concomitantly present. These results further highlight a need for reexamination, reappraisal and revision of existing guidelines with an aim to improve assessment of T2D risk.

## Supporting Information

Table S1 **Association of dichotomized WC with T2D-related traits before and after accounting for the use of lipid lowering drugs.**
(DOC)

## References

[1] Ginter E, Simko V (2010) Diabetes type 2 pandemic in 21st century. Bratisl Lek Listy 111: 134–137. 20437822

[2] Lam DW, LeRoith D (2012) The worldwide diabetes epidemic. Curr Opin Endocrinol Diabetes Obes 19: 93–96. 22262000 10.1097/MED.0b013e328350583a

[3] Osei K (2003) Global epidemic of type 2 diabetes: implications for developing countries. Ethn Dis 13: S102–106. 13677423

[4] Garber AJ (2012) Obesity and type 2 diabetes: which patients are at risk? Diabetes Obes Metab 14: 399–408. 22074144 10.1111/j.1463-1326.2011.01536.x

[5] Keller U (2006) From obesity to diabetes. Int J Vitam Nutr Res 76: 172–177. 17243079 10.1024/0300-9831.76.4.172

[6] Naser KA, Gruber A, Thomson GA (2006) The emerging pandemic of obesity and diabetes: are we doing enough to prevent a disaster? Int J Clin Pract 60: 1093–1097. 16939551 10.1111/j.1742-1241.2006.01003.x

[7] Seidell JC (2000) Obesity, insulin resistance and diabetes--a worldwide epidemic. Br J Nutr 83 Suppl 1 S5–8. 10889785 10.1017/s000711450000088x

[8] Feller S, Boeing H, Pischon T (2010) Body mass index, waist circumference, and the risk of type 2 diabetes mellitus: implications for routine clinical practice. Dtsch Arztebl Int 107: 470–476. 20644701 10.3238/arztebl.2010.0470 PMC2905837

[9] Appel SJ, Jones ED, Kennedy-Malone L (2004) Central obesity and the metabolic syndrome: implications for primary care providers. J Am Acad Nurse Pract 16: 335–342. 15455706 10.1111/j.1745-7599.2004.tb00456.x

[10] Despres JP, Lemieux I, Bergeron J, Pibarot P, Mathieu P, et al (2008) Abdominal obesity and the metabolic syndrome: contribution to global cardiometabolic risk. Arterioscler Thromb Vasc Biol 28: 1039–1049. 18356555 10.1161/ATVBAHA.107.159228

[11] Kim MK, Jang EH, Son JW, Kwon HS, Baek KH, et al (2011) Visceral obesity is a better predictor than generalized obesity for basal insulin requirement at the initiation of insulin therapy in patients with type 2 diabetes. Diabetes Res Clin Pract 93: 174–178. 21565417 10.1016/j.diabres.2011.04.009

[12] Korsic M, Fister K, Ivankovic D, Jelcic J (2011) [Visceral obesity]. Lijec Vjesn 133: 284–287. 22165197

[13] Wang Y, Rimm EB, Stampfer MJ, Willett WC, Hu FB (2005) Comparison of abdominal adiposity and overall obesity in predicting risk of type 2 diabetes among men. Am J Clin Nutr 81: 555–563. 15755822 10.1093/ajcn/81.3.555

[14] He Y, Zhai F, Ma G, Feskens EJ, Zhang J, et al (2009) Abdominal obesity and the prevalence of diabetes and intermediate hyperglycaemia in Chinese adults. Public Health Nutr 12: 1078–1084. 18986591 10.1017/S1368980008003856

[15] Mamtani MR, Kulkarni HR (2005) Predictive performance of anthropometric indexes of central obesity for the risk of type 2 diabetes. Arch Med Res 36: 581–589. 16099342 10.1016/j.arcmed.2005.03.049

[16] Schulze MB, Thorand B, Fritsche A, Haring HU, Schick F, et al (2012) Body adiposity index, body fat content and incidence of type 2 diabetes. Diabetologia 55: 1660–1667. 22349074 10.1007/s00125-012-2499-z

[17] Stevens J, Couper D, Pankow J, Folsom AR, Duncan BB, et al (2001) Sensitivity and specificity of anthropometrics for the prediction of diabetes in a biracial cohort. Obes Res 9: 696–705. 11707536 10.1038/oby.2001.94

[18] Warren TY, Wilcox S, Dowda M, Baruth M (2012) Independent association of waist circumference with hypertension and diabetes in African American women, South Carolina, 2007–2009. Prev Chronic Dis 9: E105. 22632742 10.5888/pcd9.110170 PMC3457765

[19] Wei M, Gaskill SP, Haffner SM, Stern MP (1997) Waist circumference as the best predictor of noninsulin dependent diabetes mellitus (NIDDM) compared to body mass index, waist/hip ratio and other anthropometric measurements in Mexican Americans--a 7-year prospective study. Obes Res 5: 16–23. 9061711 10.1002/j.1550-8528.1997.tb00278.x

[20] Bastarrachea RA, Kent J, Comuzzie AG (2007) [Study of the genetic component of cardiovascular risk phenotypes in a Mexican population]. Med Clin (Barc) 129: 11–13. 17570180 10.1157/13106675

[21] Bayoumi RA, Al-Yahyaee SA, Albarwani SA, Rizvi SG, Al-Hadabi S, et al (2007) Heritability of determinants of the metabolic syndrome among healthy Arabs of the Oman family study. Obesity (Silver Spring) 15: 551–556. 17372303 10.1038/oby.2007.555

[22] Voruganti VS, Lopez-Alvarenga JC, Nath SD, Rainwater DL, Bauer R, et al (2008) Genetics of variation in HOMA-IR and cardiovascular risk factors in Mexican-Americans. J Mol Med (Berl) 86: 303–311. 18204828 10.1007/s00109-007-0273-3

[23] Gao JB, Cheng JL, Ding HP, Shen MY (2011) [The disease characteristics and risk factors of type 2 diabetes mellitus in pedigrees]. Zhonghua Nei Ke Za Zhi 50: 474–477. 21781529 10.3760/cma.j.issn.0578-1426.2011.06.007

[24] MacCluer JW, Stern MP, Almasy L, Atwood LA, Blangero J, et al (1999) Genetics of atherosclerosis risk factors in Mexican Americans. Nutr Rev 57: S59–65. 10391028 10.1111/j.1753-4887.1999.tb01790.x

[25] Mitchell BD, Kammerer CM, Blangero J, Mahaney MC, Rainwater DL, et al (1996) Genetic and environmental contributions to cardiovascular risk factors in Mexican Americans. The San Antonio Family Heart Study. Circulation 94: 2159–2170. 8901667 10.1161/01.cir.94.9.2159

[26] Report of the expert committee on the diagnosis and classification of diabetes mellitus. Diabetes Care 26 Suppl 1 S5–20. 12502614 10.2337/diacare.26.2007.s5

[27] Hanley AJ, Williams K, Stern MP, Haffner SM (2002) Homeostasis model assessment of insulin resistance in relation to the incidence of cardiovascular disease: the San Antonio Heart Study. Diabetes Care 25: 1177–1184. 12087016 10.2337/diacare.25.7.1177

[28] Qu HQ, Li Q, Rentfro AR, Fisher-Hoch SP, McCormick JB (2011) The definition of insulin resistance using HOMA-IR for Americans of Mexican descent using machine learning. PLoS One 6: e21041. 21695082 10.1371/journal.pone.0021041 PMC3114864

[29] Almasy L, Blangero J (1998) Multipoint quantitative-trait linkage analysis in general pedigrees. Am J Hum Genet 62: 1198–1211. 9545414 10.1086/301844 PMC1377101

[30] Alberti KG, Eckel RH, Grundy SM, Zimmet PZ, Cleeman JI, et al (2009) Harmonizing the metabolic syndrome: a joint interim statement of the International Diabetes Federation Task Force on Epidemiology and Prevention; National Heart, Lung, and Blood Institute; American Heart Association; World Heart Federation; International Atherosclerosis Society; and International Association for the Study of Obesity. Circulation 120: 1640–1645. 19805654 10.1161/CIRCULATIONAHA.109.192644

[31] Bardini G, Dicembrini I, Pala L, Cresci B, Rotella CM (2011) Hypertriglyceridaemic waist phenotype and beta-cell function in subjects with normal and impaired glucose tolerance. Diabet Med 28: 1229–1233. 21923697 10.1111/j.1464-5491.2011.03332.x

[32] Gonzalez-Chavez A, Simental-Mendia LE, Elizondo-Argueta S (2011) Elevated triglycerides/HDL-cholesterol ratio associated with insulin resistance. Cir Cir 79: 126–131. 21631973

[33] Qiao Q, Nyamdorj R (2010) Is the association of type II diabetes with waist circumference or waist-to-hip ratio stronger than that with body mass index? Eur J Clin Nutr 64: 30–34. 19724291 10.1038/ejcn.2009.93

[34] Freemantle N, Holmes J, Hockey A, Kumar S (2008) How strong is the association between abdominal obesity and the incidence of type 2 diabetes? Int J Clin Pract 62: 1391–1396. 18557792 10.1111/j.1742-1241.2008.01805.x PMC2658023

[35] Feng RN, Zhao C, Wang C, Niu YC, Li K, et al (2012) BMI is Strongly Associated With Hypertension, and Waist Circumference is Strongly Associated With Type 2 Diabetes and Dyslipidemia, in Northern Chinese Adults. J Epidemiol 22: 317–323. 22672914 10.2188/jea.JE20110120 PMC3798650

[36] Pancoska P, Buch S, Cecchetti A, Parmanto B, Vecchio M, et al (2009) Family networks of obesity and type 2 diabetes in rural Appalachia. Clin Transl Sci 2: 413–421. 20443933 10.1111/j.1752-8062.2009.00162.x PMC4703323

[37] Tabata S, Yoshimitsu S, Hamachi T, Abe H, Ohnaka K, et al (2009) Waist circumference and insulin resistance: a cross-sectional study of Japanese men. BMC Endocr Disord 9: 1. 19138424 10.1186/1472-6823-9-1 PMC2635363

[38] Wahrenberg H, Hertel K, Leijonhufvud BM, Persson LG, Toft E, et al (2005) Use of waist circumference to predict insulin resistance: retrospective study. BMJ 330: 1363–1364. 15833749 10.1136/bmj.38429.473310.AE PMC558285

