# Correction: Waist Circumference Independently Associates with the Risk of Insulin Resistance and Type 2 Diabetes in Mexican American Families

**DOI:** 10.1371/annotation/86d53ad4-5b90-41dc-a027-07a155412f15

**Published:** 2013-11-01

**Authors:** Manju Mamtani, Hemant Kulkarni, Thomas D. Dyer, Laura Almasy, Michael C. Mahaney, Ravindranath Duggirala, Anthony G. Comuzzie, John Blangero, Joanne E. Curran

Errors occurred in Table 1 and with the reference citation brackets. In Table 1, under the heading Univariate Analysis, the B should be β. Throughout the article, the left square brackets of the reference citation are missing. 

