# Supplementary material for: Waist Circumference Independently Associates with the Risk of Insulin Resistance and Type 2 Diabetes in Mexican American Families
Source: PLoS One. 2013 Mar 11;8(3):e59153. doi: 10.1371/journal.pone.0059153 (PMC3594157; doi:10.1371/journal.pone.0059153)
Supplement: Table S1 — Association of dichotomized WC with T2D-related traits before and after accounting for the use of lipid lowering drugs. (DOC) [file pone.0059153.s001.doc]

**Table S1. Association of dichotomized WC with T2D-related traits before and after accounting for the use of lipid lowering drugs.**

| T2D-related traits | Before Accounting for lipid lowering drugs | | After accounting for lipid lowering drugs | |
| --- | --- | --- | --- | --- |
|  | β | p | β | p |
| Fasting glucose | 0.5251 | 3.81x10^-14^ | 0.5236 | 3.99x10^-14^ |
| Fasting glucose adjusted for antidiabetic drug use | 0.3659 | 2.64x10^-8^ | 0.3634 | 3.26x10^-8^ |
| Serum Insulin | 0.6025 | 5.44x10^-11^ | 0.6010 | 1.98x10^-17^ |
| Triglycerides | 0.3422 | 2.38x10^-5^ | 0.3324 | 4.16x10^-5^ |
| Total serum cholesterol | -0.0655 | 0.4252 | -0.0799 | 0.3298 |
| High density lipoprotein cholesterol (HDL-C) | -0.3142 | 0.0001 | -0.3161 | 0.0001 |
| Low density lipoprotein cholesterol directly measured (LDL-C) | -0.0839 | 0.3423 | -0.0938 | 0.2882 |
| Low density lipoprotein cholesterol fraction 1 (LDL-C f1) | -0.0803 | 0.3386 | -0.0930 | 0.2681 |
| Low density lipoprotein cholesterol fraction 2 (LDL-C f2) | -0.0736 | 0.4117 | -0.0832 | 0.3535 |

β, regression coefficient; p, significance value
